# Kinetics of high sensitive troponin T (HSTNT) after cardiac surgery

**DOI:** 10.1186/2197-425X-3-S1-A945

**Published:** 2015-10-01

**Authors:** AS Omar, S Sudarsanan, S Hanoura, H Osman, PC Sivadasan, Y Shouman, AK Tuli, R Singh, A Al Khulaifi

**Affiliations:** Hamad Medical Corporation, Cardiothoracic Surgery-Heart Hospital, Doha, Qatar; Faculty of Medicine, Critical Care Medicine, Beni_Suef, Egypt; Hamad Medical Corporation, Medical Research Center, Biomedical Statistics, Doha, Qatar

## Introduction

Perioperative myocardial infarction (PMI) in the setting of cardiac surgery represent a considerable risk to patients with significant mortality and morbidity. The role of highly sensitive cardiac troponin T (hsTnT) has not been evaluated in the setting of cardiac surgery, instead Cardiac Troponin T (TnT) and Creatine Kinase MB (CK-MB) are commonly used for the diagnosis of PMI [1].

## Objectives

Assess the diagnostic accuracy and kinetics of hsTnT in the setting of cardiac surgery and to define the serum level at which PMI can be diagnosed in comparison to CK-MB.

## Methods

Single center prospective observational study conducted over a 2 year period. Data from all patients undergoing cardiac surgery was analysed. Patients with chronic renal impairment, sepsis, or preexisting high level of hsTnT were excluded. The primary outcome was the diagnosis of PMI as defined by a specific level of CK-MB. The secondary outcome measures were the length of mechanical ventilation (LOV), length of stay in the intensive care unit (LOS_ICU_), and length of hospitalization. Receiver operating curve (ROC) was used to test the validity of hsTnT as a marker of PMI. Based on the Third universal definition of PMI patients were divided into two groups: Group I: no PMI; Group II: PMI (2.17% of the study population).

## Results

Data from 413 patients with mean age (± SD) of 54.9 (10.9) years were analysed. Both groups were matched for age, body mass index (BMI), prevalence of diabetes mellitus, serum creatinine, Euroscore, aortic cross clamp and cardiopulmonary bypass time, and total length of anesthesia. 9 patients fulfilled the diagnostic criteria of PMI, where 41 patients were identified with a 5-fold increase in the CK-MB (≥120 U/L). Using ROC analysis, the hsTnT level of 3466 ng/L or above showed 90% sensitivity and 90% specificity for diagnosis of PMI and the level of 2309 ng/L showed 80% sensitivity and 86% specificity for suspicected myocardial injury. LOV and LOS_ICU_ were significantly higher in Group II.

## Conclusions

The hsTnT levels detected here paralleled those of CK-MB and a cut-off level of 3466 ng/L could be diagnostic of PMI. Further studies are required to validate this finding. Secondary outcome measures in patients with PMI (i.e., LOV and LOSICU) were significantly prolonged.

## Grant Acknowledgment

To all members of cardiothoracic surgery department and medical research center, Hamad medical corporation, Qatar

**Table 1:** Table 1

Variable	Group I(n = 404)	Group II (n = 9)	P-value
LOV (minutes)	422 ± 211	1567 ± 597	0.000
LOSICU (hours)	61.6 ± 9.8	1567 ± 597	0.05
LOShosp (days)	12.18 ± 2.5	408.4 ± 70.5	0.000
POAF	14 (3.4%)	5 (55.6%)	0.01
AKI	120 (29%)	7 (77.8%)	0.04
Re-admision ICU	9 (2.2%)	2 (22.2%)	0.01
Re-exploration	30 (7.4%)	2 (22.2%)	0.001
Inhospital mortality	9 (2.2%)	5 (55.6%)	0.01
LOV length of mechanical ventilation, LOSICU ICU length of stay, LOShosp Hospital length of stay, POAF post-operative atrial fibrillation, AKI acute kidney injury			

*[Clinical outcome in both groups]*
